# Unsafe water, sanitation and hygiene: a persistent health burden

**DOI:** 10.2471/BLT.23.290668

**Published:** 2023-09-01

**Authors:** Bruce Gordon, Sophie Boisson, Richard Johnston, David J Trouba, Oliver Cumming

**Affiliations:** a Department of Environment, Climate Change and Health, World Health Organization, Avenue Appia 20, 1211 Geneva 27, Switzerland.; b Department of Disease Control, Faculty of Infectious Disease, London School of Hygiene & Tropical Medicine, London, England.

A World Health Organization (WHO) study estimates that 1.4 million deaths could be prevented each year through improving access to safely managed water, sanitation and hygiene services.^1^^,^^2^ While modest progress has been made towards meeting targets of the sustainable development goal (SDG) 6, which calls for ensuring the availability and sustainable management of water and sanitation for all,^3^ progress is insufficient to achieve the SDG ambition of universal access for all by 2030. In 2022, 2.2 billion people lacked safely managed drinking water at home, 3.5 billion people did not have safely managed sanitation and 2 billion people could not wash their hands with soap and water at home.^3^ Furthermore, since mid-2021, several low- and middle-income countries saw a surge in cholera outbreaks, including countries unaffected in decades.^4^

In March 2023, the United Nations (UN) convened its first all-UN conference on water in half a century to sound a call to action to all governments to accelerate national efforts towards achieving SDG 6. However, this call might be difficult to heed because in a 2022 WHO survey, 95 out of 113 governments reported insufficient funding to implement their water, sanitation and hygiene plans and strategies.^5^ Still, WHO and the United Nations Children’s Fund (UNICEF) report that since 2015, the estimated global coverage has increased for basic water (from 88% to 91%), basic sanitation (from 73% to 81%) and basic hygiene (from 67% to 75%) services.^3^

This progress still falls short of the SDG 6 threshold of safely managed services, which requires safe water to be available at home when needed, and for sanitation to include not only a toilet but a system to treat and manage excreta. The burden of disease analyses of water, sanitation and hygiene corroborate that the transition to safely managed services results in much greater health gains.^2^^,^^6^

Consensus is growing that delivering safely managed water and sanitation services requires significant strengthening of government systems, professionalization of service delivery and major increases in investment. The scale of reform required may disincentivize the necessary action, yet some countries have rapidly improved water, sanitation and hygiene with public investment, responsible management, clear data and accountable governance. India’s Jal Jeevan Mission, for example, is an unprecedented investment in scaling up household water connections. As with India’s earlier sanitation-focused initiative, where high-level political support catalysed a mass movement that engaged government, households and the private sector,^7^ the Jal Jeevan Mission shows that considerable gains can be achieved in a short time and in full alignment with the SDGs.

Much can be done to better target the existing government budgets and limited external support. One key action is to prioritize water, sanitation and hygiene programming in national health strategies, recognizing its cost-effectiveness to prevent disease, reduce health-care costs and improve productivity. For example, the world’s 46 least-developed countries could have universal handwashing facilities by 2030 if governments invested less than 1 United States dollar per person per year in hand hygiene.^8^

Second, using health surveillance data to inform water, sanitation and hygiene service delivery in disease hotspots will be important to identify high-priority areas and inform greater collaborative actions. Through the Expanded Special Project for Elimination of Neglected Tropical Diseases, WHO and its partners have developed water, sanitation and hygiene coverage and neglected tropical diseases endemicity overlay maps at the implementation unit level for 47 African countries. 

A third key action, and the basis of the WHO water, sanitation and hygiene strategy 2018–2025,^9^ is to incrementally apply health-based norms, standards and regulations to water, sanitation and hygiene services. These norms, such as those articulated in WHO’s guidelines for drinking water quality^10^ and sanitation and health,^11^ support standard-setting and regulations at the national level.

Yet two challenging realities exist regarding water, sanitation and hygiene-related diseases.

The first is that without immediately scaling up investment and strengthening existing systems, the attributable disease burden will change little in the years ahead.

The second is the urgent need to provide at least basic services to those who still lack them, including vulnerable populations in rural areas. Otherwise, cholera outbreaks and chronic neglected tropical diseases will continue.

In the face of climate change, urbanization and the need to prepare for pandemics, WHO and UNICEF have issued a joint statement urging governments to save lives, improve health outcomes and prevent disasters through action on water, sanitation and hygiene.^12^ The statement’s sentiments were echoed in the voluntary commitments from governments, businesses and civil society entities participating in the UN 2023 Water Conference and the subsequent High-Level Political Forum in July 2023 to accelerate progress towards SDG 6.
